# A portable assist-as-need upper-extremity hybrid exoskeleton for FES-induced muscle fatigue reduction in stroke rehabilitation

**DOI:** 10.1186/s42490-019-0028-6

**Published:** 2019-11-19

**Authors:** Ashley Stewart, Christopher Pretty, Xiaoqi Chen

**Affiliations:** Mechanical Engineering, University of Canterbury, 20 Kirkwood Ave, Upper Riccarton, Christchurch, 8041 New Zealand

**Keywords:** Rehabilitation, Functional electrical stimulation, Hybrid exoskeletons, Muscle fatigue

## Abstract

**Background:**

Hybrid exoskeletons are a recent development which combine Functional Electrical Stimulation with actuators to improve both the mental and physical rehabilitation of stroke patients. Hybrid exoskeletons have been shown capable of reducing the weight of the actuator and improving movement precision compared to Functional Electrical Stimulation alone. However little attention has been given towards the ability of hybrid exoskeletons to reduce and manage Functional Electrical Stimulation induced fatigue or towards adapting to user ability. This work details the construction and testing of a novel assist-as-need upper-extremity hybrid exoskeleton which uses model-based Functional Electrical Stimulation control to delay Functional Electrical Stimulation induced muscle fatigue. The hybrid control is compared with Functional Electrical Stimulation only control on a healthy subject.

**Results:**

The hybrid system produced 24° less average angle error and 13.2° less Root Mean Square Error, than Functional Electrical Stimulation on its own and showed a reduction in Functional Electrical Stimulation induced fatigue.

**Conclusion:**

As far as the authors are aware, this is the study which provides evidence of the advantages of hybrid exoskeletons compared to use of Functional Electrical Stimulation on its own with regards to the delay of Functional Electrical Stimulation induced muscle fatigue.

## Background

Stroke is the second largest cause of disability worldwide after dementia [1]. Temporary hemiparesis is common among stroke survivors. Regaining strength and movement in the affected side takes time and can be improved with the use of rehabilitation therapy involving repetitive and function-specific tasks [2]. Muscle atrophy is another common issue that occurs after a stroke due to lack of use of the muscle. For each day a patient is in hospital lying in bed with minimal activity approximately 13% of muscular strength is lost (Ellis. Liam, Jackson. Samuel, Liu. Cheng-Yueh, Molloy. Peter, Paterson. Kelsey, Lower Limb Exoskeleton Final Report, unpublished). Electromechanically actuated exoskeletons offer huge advantages in their ability to repetitively and precisely provide assistance/resistance to a user. However electromechanical actuators which provide the required forces are often heavy in weight and have high power requirements which limits portability. Furthermore, muscle atrophy can only be prevented by physically working the muscles either through the patient’s own volition or the use of Functional Electrical Stimulation (FES).

FES is the application of high frequency electrical pulses to the nerves or directly to the muscle belly in order to elicit contractions in the muscle. FES devices are typically lightweight and FES is well suited to reducing muscle atrophy in patients with no or extremely limited movement. The trade off to this is that precise control of FES is extremely difficult and controlling specific, repetitive, and functional movement is not easily accomplished. Furthermore, extended use of FES is limited by the introduction of muscle fatigue caused by the unnatural motor unit recruitment order [3]. The forces required for large movements, such as shoulder abduction, are too great to be provided by the use of FES which is much better suited to smaller movements such as finger extension [4, 5]. Some patients also find the use of FES painful.

Combining the use of FES and an electromechanical actuator within an exoskeleton can potentially overcome the limitations of each individual system. Despite the potential advantages of hybrid exoskeletons, so far only limited studies have been done on their effectiveness. A recent review was conducted into upper-extremity hybrid exoskeletons [6] which highlighted the advantages hybrid exoskeletons (exoskeletons which combine FES with an actuator) have with regards to improving the precision of FES induced movements. However, little attention has been given towards reduction and management of FES-induced fatigue. FES control systems used for upper-extremity hybrid exoskeletons simply manually ramp up stimulation intensity when fatigue is observed.

This work describes the design and testing of an assist-as-need upper-extremity hybrid exoskeleton which uses model-based control of FES with a focus on reducing FES-induced muscle fatigue. The control system is described in Section “Theory”, and the results are presented in Section “Results”. A discussion of the results is given in Section “Discussion”. Conclusions are summarised in Section “Conclusion”. Methods, physical structure of the exoskeleton, and the sensing system is described in Section “Material and methods”.

## Theory

It is highly desirable in stroke rehabilitation robotics that a robot or exoskeleton be capable of performing assistance-as-needed. This way the patient is encouraged to make the effort to achieve movement rather than learning to rely on the robot to perform the movement [7–9]. Appropriately timed action is more important than strength for functional gains, however repetitive practice which builds strength without specific functional application can still help to diminish impairment [10]. The ability of rehabilitation robots to adapt to different users and even to the same user on different days or throughout the same session is also highly important with regards to minimising set-up time and cost of rehabilitation [9]. In general the robot should aid and encourage but not limit the movement of the patient [9, 11]. Above all else the robot should pose no harm to the user or nearby individuals.

To implement the concept of assist-as-need there are two important features which are desired:

1. The assistance provided from the FES and motor should be the minimum which the patient requires to perform the movement at a given time.
2. The FES should perform the bulk of the movement which the patient is physically unable to. This ensures that most of the movement performed requires effort form the patient’s muscles and thus improves muscular strength.

In the system proposed here the angle of the arm can be affected and controlled by three different inputs; volitional movement from the subject, FES-induced movement, and rotation of the motor. Any one of these on their own could potentially produce the desired angle. However to achieve the two defined desires, there is a necessary hierarchy of control.

**Fig. 1:**
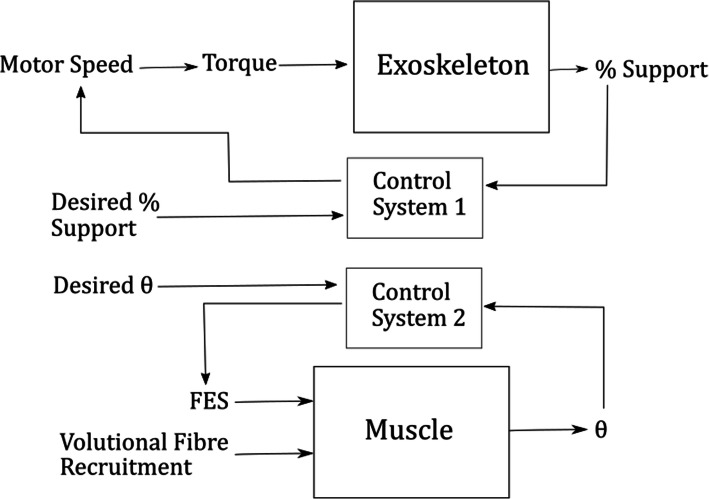
High Level Control of the Hybrid System

The control system may in general be clearly divided into at least two control systems, each related to a different output variable which can be considered independently (Fig. 1). There is one situation however, where this is not the case. This situation would occur if neither the FES nor the user were able to provide sufficient torque to produce the movement. In this type of situation the motor should provide positive active assistance for the user and the control for the motor would be based on the angle rather than the measured support. It is important to note that the assistance that the motor provides is purely for flexion. The motor may slow the rate at which the arm extends but it cannot pull the arm down faster than gravity.

Section “Setup” will describe the system set-up process. Sections “Motor” to “FES Gain (k)” will present the individual control systems for each of the four variables; the arm angle, the % support, the desired % support, and the overall gain for the FES, respectively.

### Setup

Previous work [12] investigated the performance of new linear model for FES control. This model is described by Eq. 1.

1 $$ \Delta  \uptheta =\mathrm{k}\left({\mathrm{v}}_{\mathrm{g}}\Delta  \mathrm{v}+{\mathrm{pw}}_{\mathrm{g}}\Delta  \mathrm{pw}+{\mathrm{f}}_{\mathrm{g}}\Delta  \mathrm{f}\right) $$

Where:

θ is the elbow angle in degrees

v is the voltage in volts

pw is the pulse-width in microseconds (full pulse length = positive + negative portions)

f is the frequency in Hertz

k is the overall gain

v_g_= 14, voltage gain

pw_g_ = 0.15, pulse-width gain

f_g_ = 0.22, frequency gain

The model performed well for different subjects and only the threshold voltage and the overall gain needed to be found for the subject. As described in Section “Material and Methods”, to measure the support percentage, knowledge of the user’s arm weight is also required. Because this system is adaptive it is possible to initially estimate the value of k as something conservative (higher rather than lower so the system starts with a small stimulation intensity) and have the system recalculate k at run time. Thus, there are only two parameters which must be obtained during setup. These are obtained as follows:

1. The user is instructed to relax their arm so that the palm faces the user (parallel to the sagittal plane) with fingers pointed down. Once the user is relaxed the system is switched on.
2. At the beginning of setup the motor rotates the arm to 90°. Five measurements each are taken of the angle and torque. These readings are averaged and used to calculate the weight of the arm under the assumption that the arm and exoskeleton are a point mass at distance 0.13 m from the elbow. The motor then lowers the arm back to 0°.
3. The voltage threshold test is conducted. Stimulation is applied at a frequency of 30.5 Hz, and pulse-width of 200 μs. Voltage steps are applied in increments of 0.5 V starting at 10 V. Each step is applied for a duration of 3 s and the peak arm response is recorded in degrees. When a step results in a peak arm angle of 20° the voltage threshold test is complete and the input voltage is recorded and defined as the threshold voltage. In between each step if a 20° angle has not been achieved then then stimulation is turned off for a duration of 3 s before the next step is applied. This short rest is to prevent the arm getting used to the stimulation which would affect the voltage threshold (more stimulation would be required to achieve a given angle).

The entire system takes less than 6 min for a complete set up including attachment of the exoskeleton and electrodes. Once the setup has completed, the control system runs on the right arm in response to a desired arm angle based on the position of the left arm. Control of this system is described in sections “Motor to FES Gain (k)”.

### Motor

When the desired support is less than 100% the motor speed is set using proportional-differential (PD) control based on the support error. When the desired support is 100% the motor speed is set using proportional-derivative (PD) control based on the angle error for errors larger than 5°. If the angle error is negative and greater than 20° then the motor will lower the arm at the maximum speed. This last state is to ensure that the motor does not impede movement of the user. Due to the setup of the pulley and cable system the motor cannot physically impede movement of the user when raising the arm but could potentially impede movement during lowering.

### Fes

Control of FES is performed using the model described by Eq. 1. Updating of the FES parameter inputs is only performed if the desired angle has changed by more than 5° or if the time since the last update has exceeded 0.5 s. This is to give the muscle time to respond to the stimulation. These values were experimentally found to be suitable while still allowing for a faster response from the FES than from the motor. When the FES is updated the left side of Eq. 1 is equal to the angle error. Equation 1 is then used to calculate the required change in each input parameter so that each parameter contributes the same change in angle.

### Desired support

The control of the desired amount of support is performed based on the angle error over time. In general if the error over time is consistently positive then desired support should increase. If, on the other hand, the error is consistently negative then support should decrease. In general the desired support should not respond too quickly to errors in the angle and it should ignore any large short term errors. Thus rather than use the average error over time a median filter of length 50 is used. A measurement of the angle error is taken every 0.5 s.

If the median error over the last 25 s is within 5° then no changes are made to the desired amount of support. If the median error is positive and larger than 5° the desired support is increased. If the median error is negative and larger than 5° the desired support is decreased. The previous median error is also used to calculate how much the desired support should be changed. If there has been a change in both the desired support and the median error then those values are used to calculate the new support using Eq. 2. If there has not been a change in the desired support or the median angle error then the desired support is changed by 1% for every 1° median angle error, for median angle errors greater than 5°. Regardless of what the median error is, if all of the FES parameters used for the current step are applied at their maximum values the desired support will be increased by 20%. To prevent rapid changes in the desired support the maximum change is limited to 20% every 0.5 s.

2 $$ \mathrm{S}\left(t+0.5\right)=S(t)+\left[\ \frac{\left[\overset{\sim }{e_{\theta }}\left(t+0.5\right)-\overset{\sim }{e_{\theta }}(t)\right]\ }{\overset{\sim }{e_{\theta }}(t)-\overset{\sim }{e_{\theta }}\left(t-0.5\right)}\ x\ \left[S(t)-S\left(t-0.5\right)\right]\ \right] $$

Where:

S is the desired % support

$\overset{\sim }{e_{\theta }}$ is the median angle error

t is the current time

$\overset{\sim }{e_{\theta }}\left(t+0.5\right)$ is the desired angle error at t + 0.5 s which is set equal to 0°

### FES gain (k)

Every 0.5 s Eq. 1 is used to calculate the overall gain (k) using the measured right arm angle (minus the 20° threshold) and FES parameters (minus their respective thresholds). If the right arm angle is greater than 20°, the input parameters are greater than their threshold values, and thus the overall gain is positive then the calculated value for the overall gain is added to an array. The array contains the last 50 calculations for the overall gain. Each 0.5 s the median value for the overall gain is retrieved from the array and, after checking limits, is set as the new overall gain value used to calculate the future FES parameter step sizes given a desired arm angle change. The change in the overall gain is limited to plus or minus 0.2 each 0.5 s.

## Results

Ten tests were conducted on Exoskeleton using a healthy 27-year old female subject with different initial values for the overall gain and desired assistance. Selected plots of the test results are displayed in Sections “Test 2 – 2 Minutes of Hybrid Control, k = 1, Assist = 0 %” to “Summary of Results for All Tests”, and Figs. 2, 3, 4, 5, 6, 7, 8 and 9. Subsection “Summary of Results for All Tests”, contains a summary of all the results. The test details are summarised in Table 1, and the results for each tests are summarised in Table 2 in Subsection “Summary of Results for All Tests”. Ethical approval for testing was granted by the University of Canterbury Human Ethics Committee.

**Table 1:** Initial Parameters, Control Scheme, and Test Length for Tests Conducted Using the Hybrid Exoskeleton on one Healthy Individual

Test Number	Initial FES gain (k)	Initial Assist (%)	Test Time (mins)	Volitional Movement	Control
1	1	50	2	No	Hybrid
2	1	0	2	No	Hybrid
3	1.8	50	2	No	Hybrid
4	1.8	0	2	No	Hybrid
5	1	50	2	Yes	Hybrid
6	10	0	6	No	FES Only
7	10	0	6	No	Hybrid
8	10	0	2	No	Hybrid
9	1	0	6	No	Motor Only
10	1	100	2	No	Motor Only, no assist-as-need

**Table 2:** Summary of Exoskeleton Test Results, Initial Parameters, Control Scheme, and Test Length for Tests Conducted Using the Hybrid Exoskeleton on one Healthy Individual. RMSE = Root Mean Square Error

Test n.o.	Initial k	Initial Assist (%)	Test Time (mins)	Volitional Movement	Control	Final k	Median Error	Average Error	v_thresh_(v)	RMSE
1	1	50	2	No	Hybrid	0.6	−5.2	− 0.6	16	27.9
2	1	0	2	No	Hybrid	0.6	4.6	0.8	17	21.2
3	1.8	50	2	No	Hybrid	0.4	−3.4	−0.8	18.5	24.9
4	1.8	0	2	No	Hybrid	0.4	0.2	0.4	20	28.6
5	1	50	2	Yes	Hybrid	1	−8.1	−9.6	20.5	12.5
6	10	0	6	No	FES Only	0.2	27.3	25	20.5	42.9
7	10	0	6	No	Hybrid	0.4	−0.9	−4	22.5	29.7
8	10	0	2	No	Hybrid	0.4	4.3	6.8	19.5	39.7
9	1	0	6	No	Motor Only	–	20.7	25.5	–	51.1
10	1	100	2	No	Motor Only,no assist-as-need	–	−9.1	−8.8	–	21.8

All tests, except Test 5, were conducted with the user providing no volitional input from their right arm. Test 5 involved the user moving both arms volitionally together in a mirroring pattern. Test 6 used FES only with no assistance form the motor. Test 9 and 10 used only the motor and no FES. Only Test 10 did not perform assist-as-need. The tests are listed in the order they were conducted and only short rests (a few minutes) were taken between each test. All tests were conducted on the same day. A discussion is given for the tests and results in Section “Discussion”, following the figures. Some mechanical issues were had following Test 7, resulting in a longer rest time (about 30–60 min) prior to Test 8. Any effects this had are discussed in Section “Discussion”.

The first figure in each test subsection displays the desired angle (angle of the left arm, input) and measured angle (angle of the right arm, output) during the test. In cases where there is a second figure this shows the change in the desired support and the change in the gain during the test in response to the assist-as-need control scheme.

### Test 2–2 minutes of hybrid control, k = 1, Assist = 0%

**Fig. 2:**
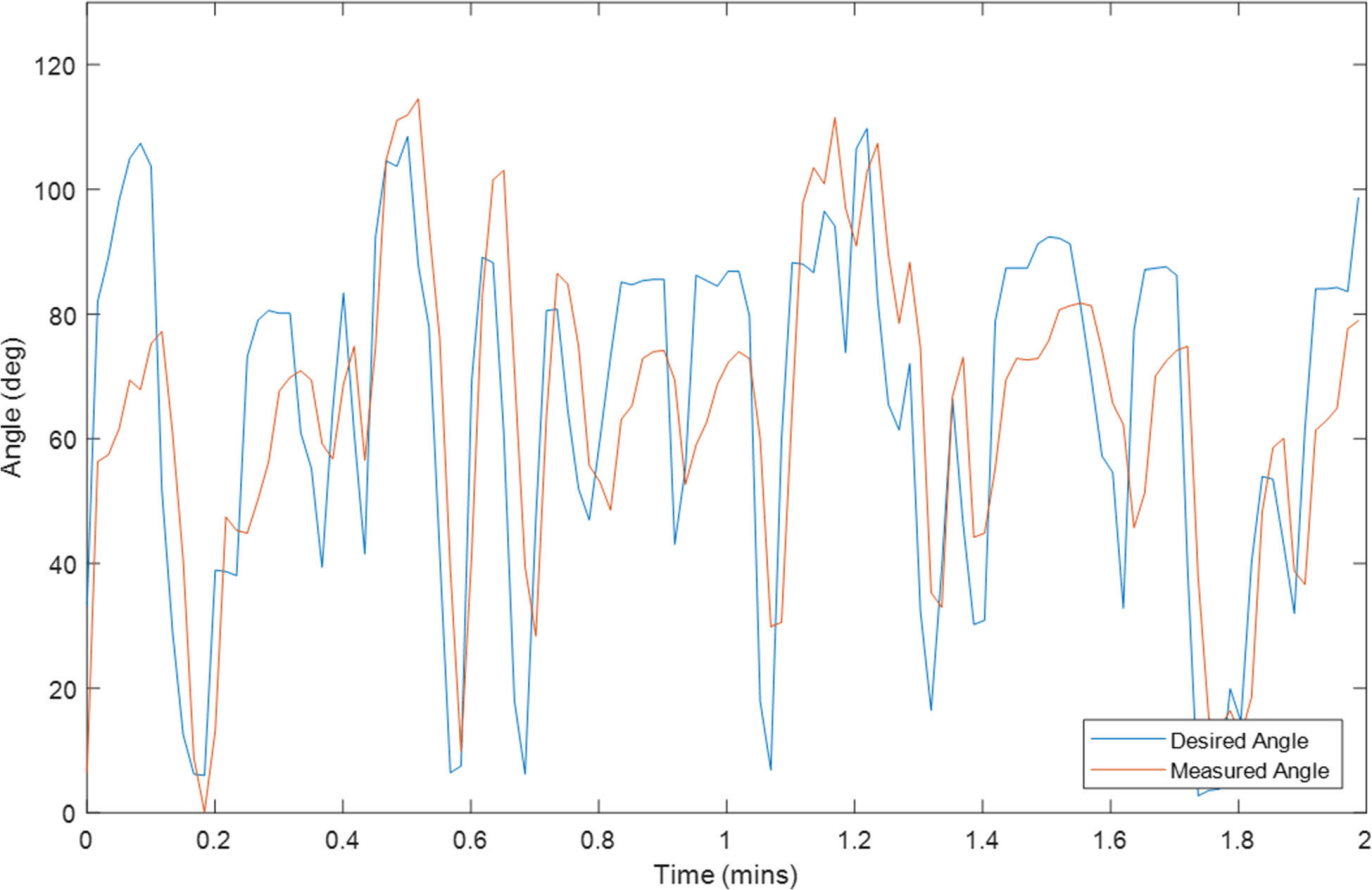
Right Arm Angle (Orange) and Left Arm Angle (Blue) during Test 2

### Test 5–2 minutes of hybrid control with volitional movement, k = 1, Assist = 50%

**Fig. 3:**
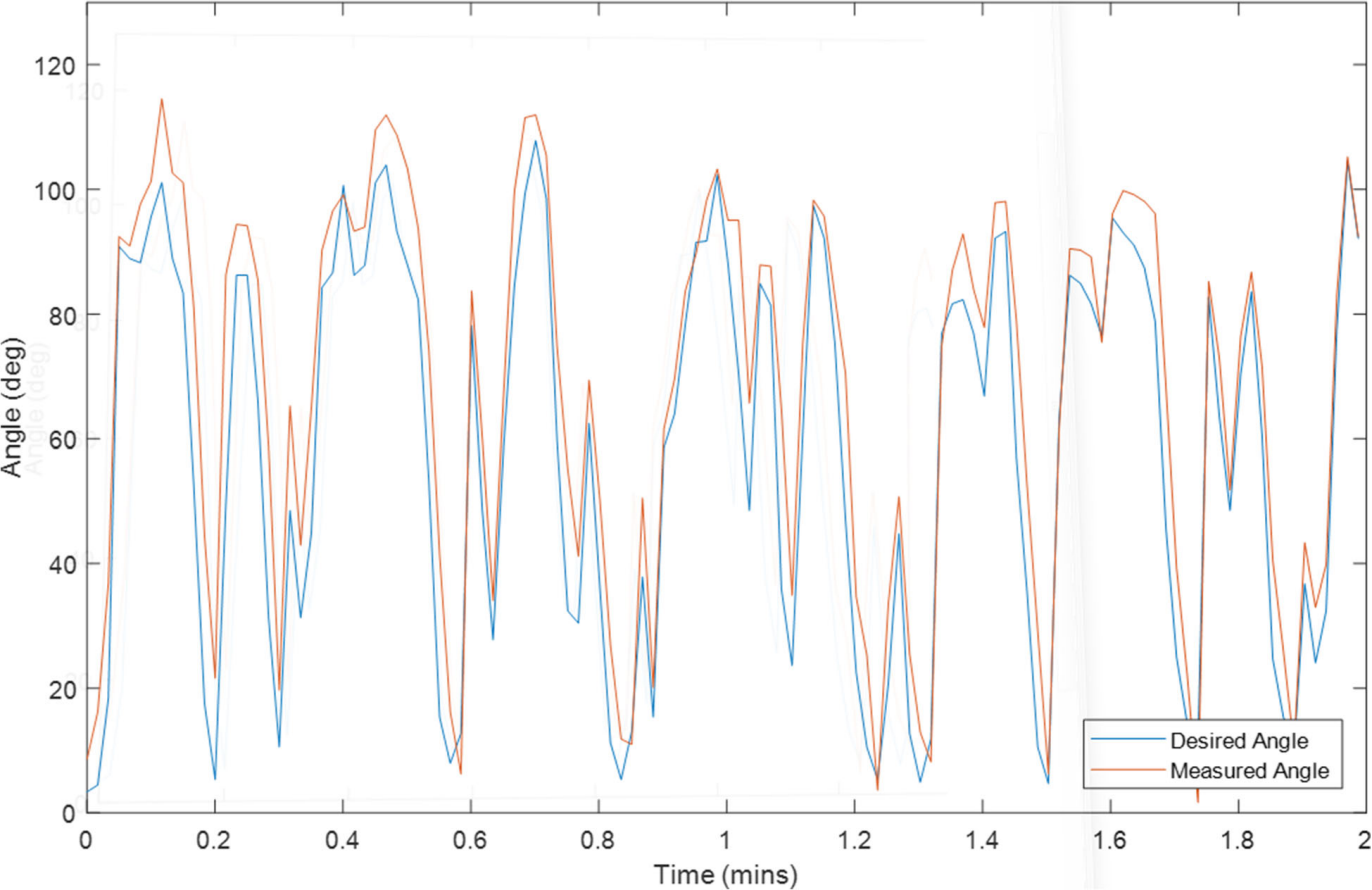
Right Arm Angle (Orange) and Left Arm Angle (Blue) during Test 5

**Fig. 4:**
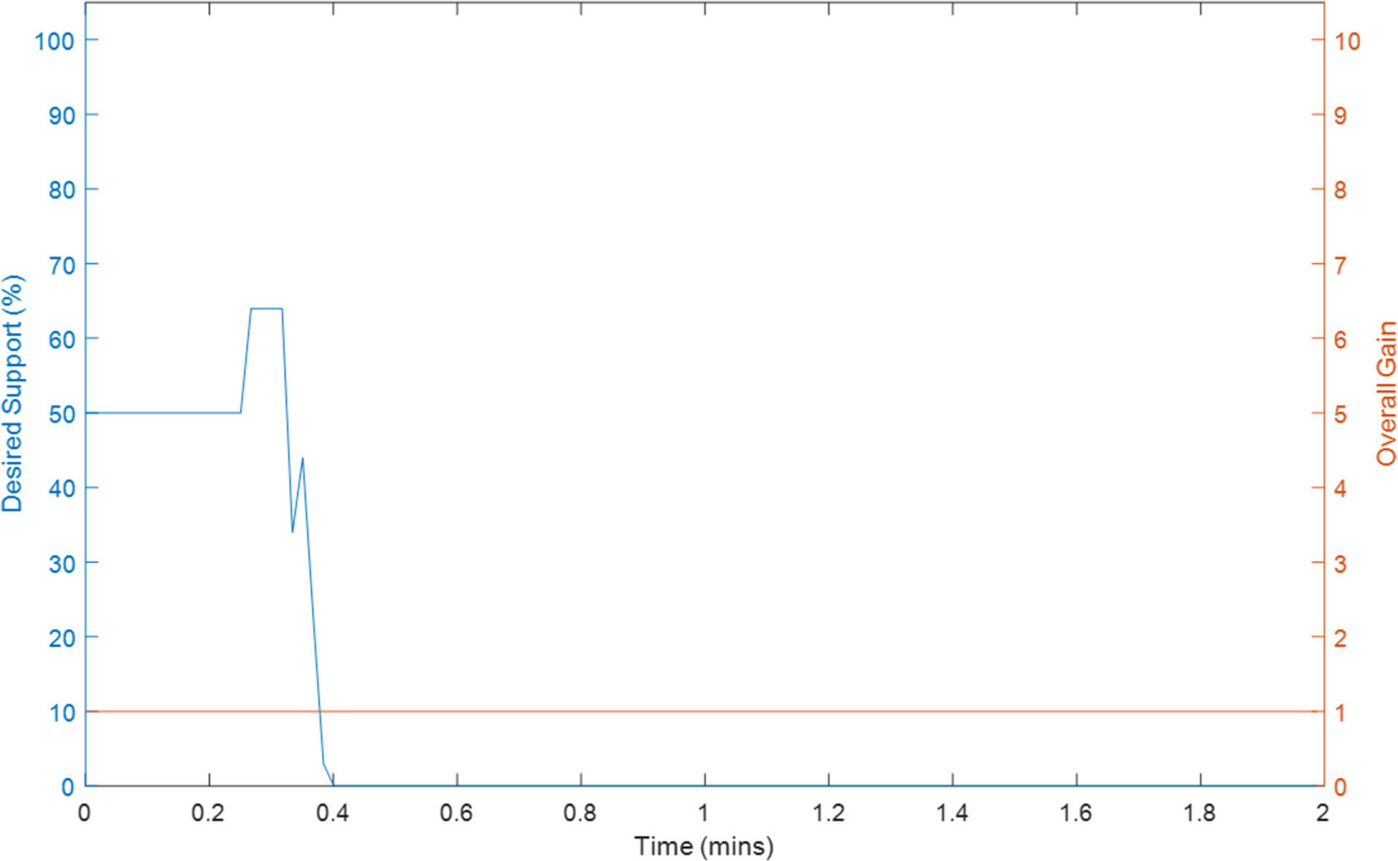
Variation in Overall Gain (Orange) and Desired Support (Blue) during Test 5

### Test 6–6 minutes of FES control, k = 10, Assist = 0%

**Fig. 5:**
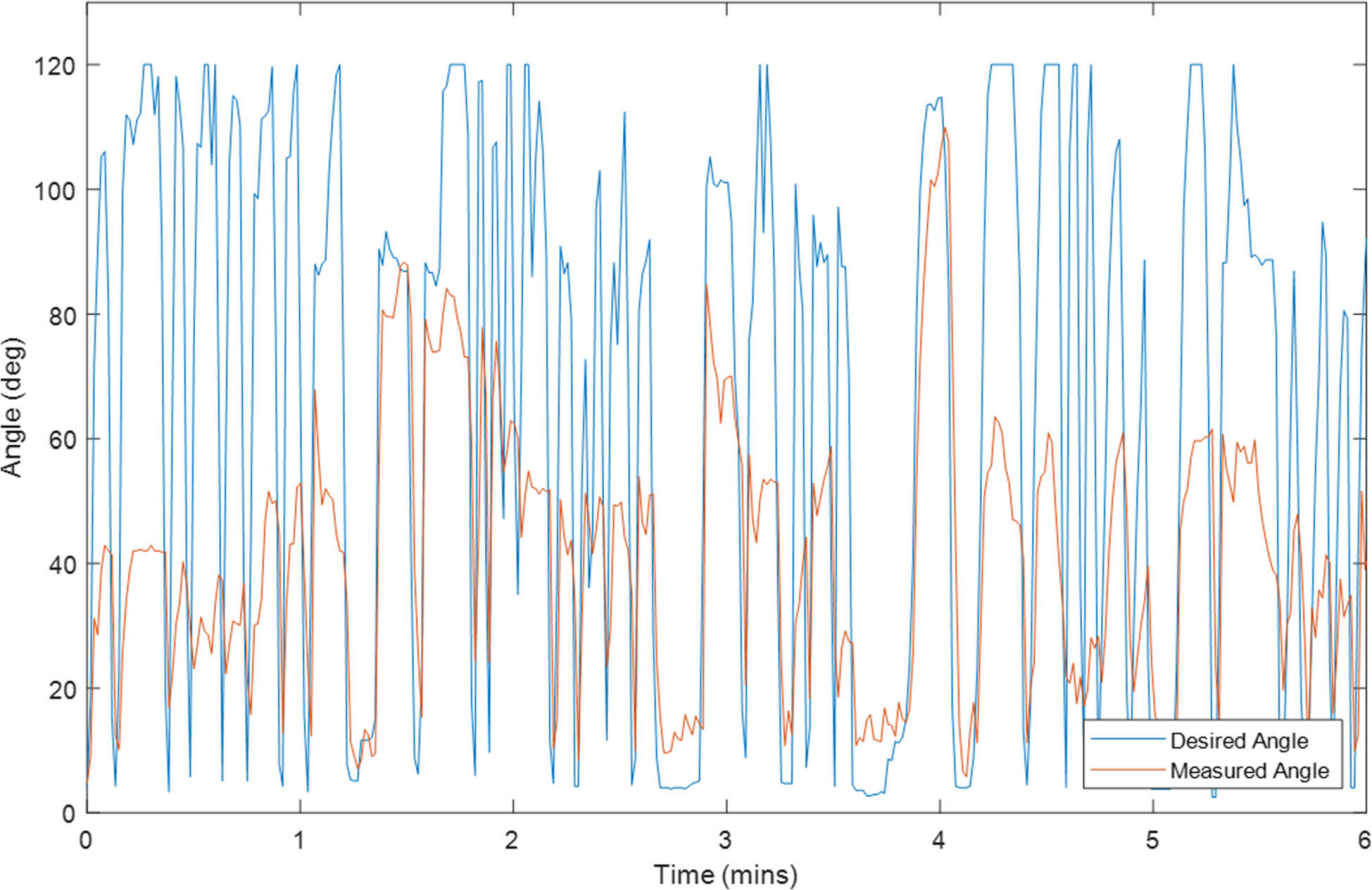
Right Arm Angle (Orange) and Left Arm Angle (Blue) during Test 6

### Test 7–6 minutes of hybrid control, k = 10, Assist = 0%

**Fig. 6:**
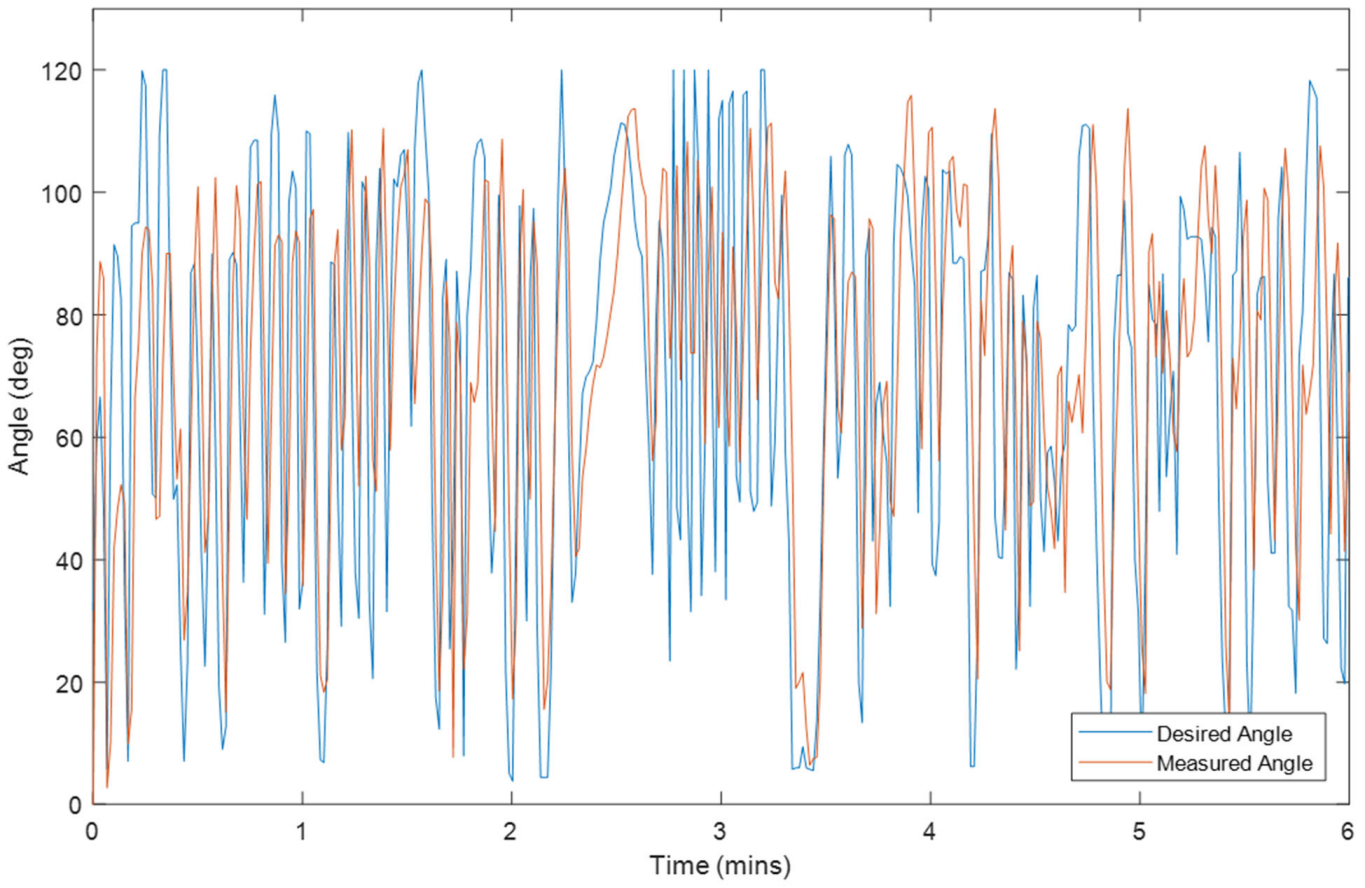
Right Arm Angle (Orange) and Left Arm Angle (Blue) during Test 7

### Test 10–2 minutes of motor control without assist-as-need, k = 1, Assist = 100%

**Fig. 7:**
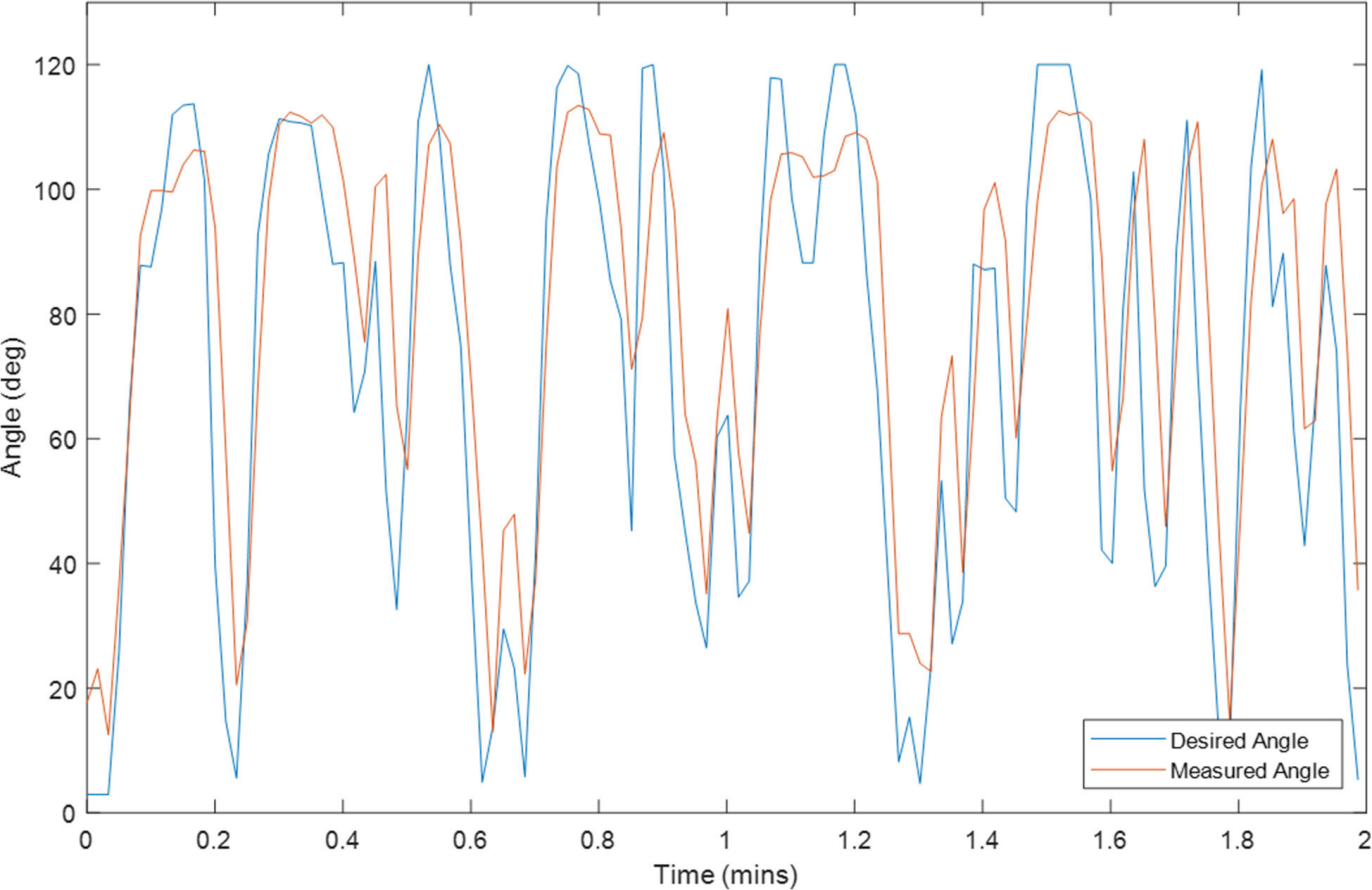
Right Arm Angle (Orange) and Left Arm Angle (Blue) during Test 10

### Summary of results for all tests

**Fig. 8:**
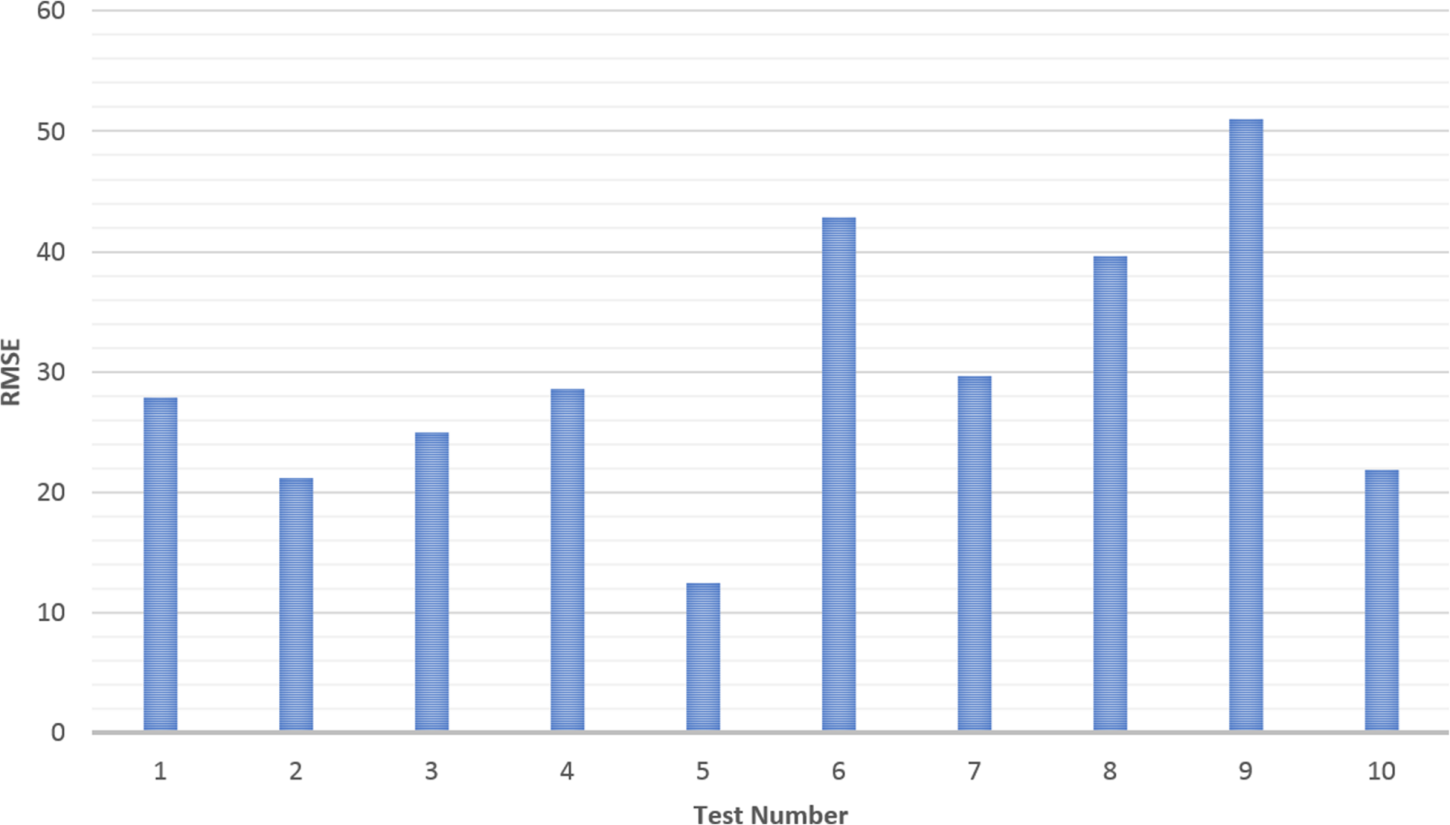
Root Mean Square Error (RMSE) for Each Test

**Fig. 9:**
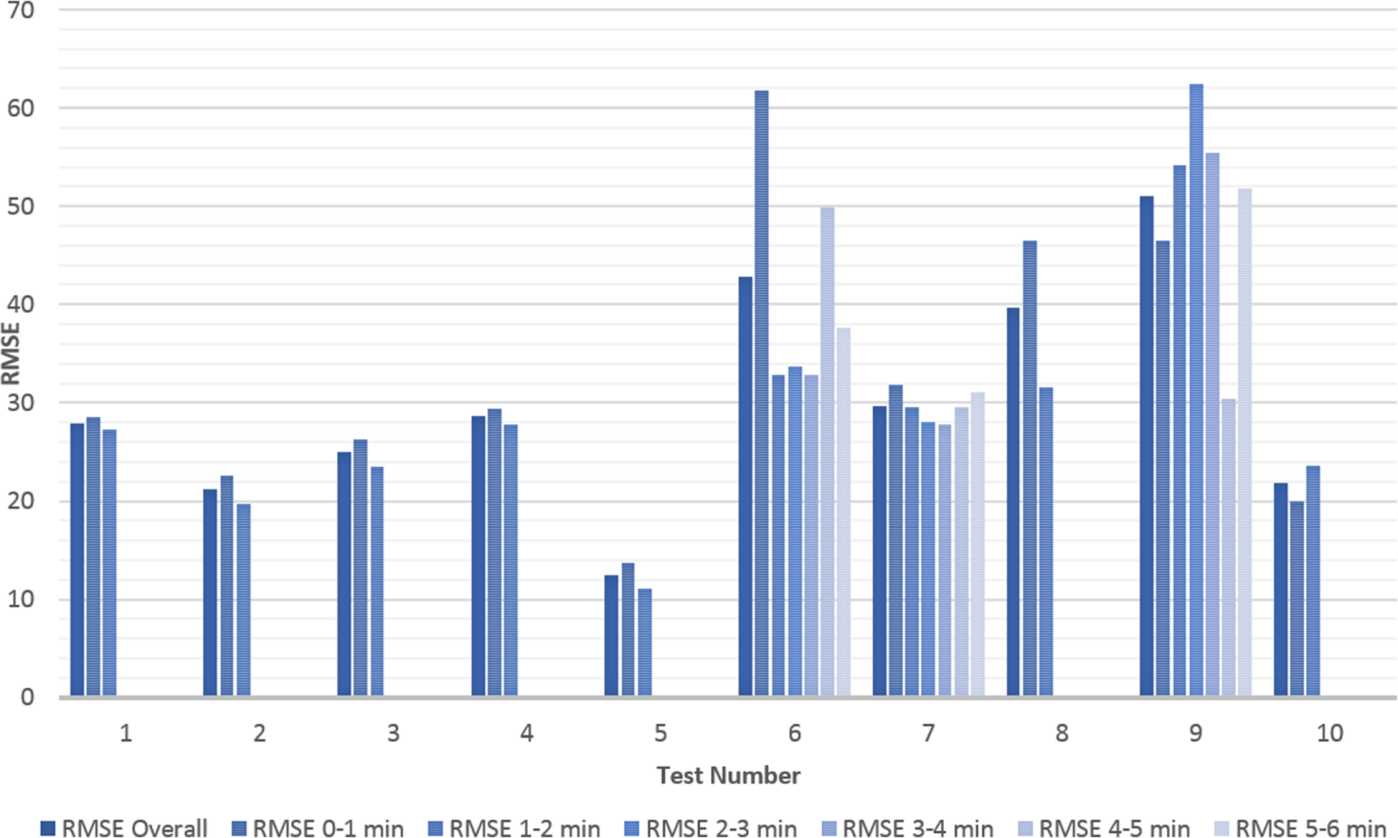
Change in Root Mean Square Error (RMSE) during Each Test

## Discussion

The results for the tests are given in Table 2 and Fig. 8 and Fig. 9 at the end of the previous section. It is important to note that the input reference angle trajectory was not the same for every test thus these results should only be used to give a general high-level performance comparison. It should also be noted from the angle comparison plots that the right-arm rest angle appeared to be slightly higher than that of the left arm. This is likely due to the fact that the left arm was controlled volitionally the entire time whereas the right arm was in a relaxed state. When the arm is relaxed it was observed to not often rest exactly at 0° but rather a little higher and the elbow has a slight bend. Thus, the controlling left arm would be physically held at 0° and the right-arm would settle slightly above 0° in response. This will result in a slight shift in the median and average angle error towards the negative, and an increase in the magnitude of the Root Mean Square Error (RMSE).

This is likely why at initial glance the volitional test (Test 5) and the motor only without assist-as-need test (Test 10) appear to have larger average and median errors than the hybrid tests. It is expected that these two tests should produce the smallest errors. That said, from the plots for these two tests (Figs. 3 and 7) it can be seen that there is also some error at the peaks as well as at the troughs so not all of error can be attributed to the rest angle. Furthermore, when comparing the RMSE instead of the median and average errors, the volitional test (Test 5) does perform the best, as expected, with the lowest RMSE value. It is important to note that no time delay was considered when comparing the desired angle with the measured angle, thus the response time should cause a larger measured error for all tests compared with the volitional test, during which the movement was conducted simultaneously. It is worth noting that even volitional movement for a healthy subject without a time delay does not produce perfect tracking.

Overall, the first four Hybrid Control Tests (Tests 1–4) performed similarly to that of the motor only control (Test 10) with similar sized error measurements across the board. There was some variation in performance among the Hybrid control tests depending on the initial test parameters (k and desired assistance), variations in test time, and variations in fatigue, however no noticeable trend was observed and the differences were not large. The difference in RMSE between the best and worst of the first four hybrid control tests was 7.44 degrees.

There are three tests which stand out as having large errors. These are Test 6, 8 and 9. Test 6 is the FES only control test and given that the difficulties with performing large movements with FES and FES-induced fatigue are well known problems for FES, it is not surprising that Test 6 has the worst performance. The results for Test 8 and 9 are less expected and will be discussed later on in this section.

Due to all of these tests being conducted on the same day and one after another it is expected that the arm will be more fatigued for the later tests. This is backed up by the general increase seen for the voltage threshold (v_thresh_) and the general decrease in the overall gain (k). It is for this reason that the 6 min FES only tests (Test 6) was conducted prior to the 6 min long Hybrid test (Test 7). If the hybrid control is able to reduce the impact of FES-induced fatigue then it is expected that the performance of Test 7 should be better than that of Test 6, however it is also expected that a good performance would be harder to achieve in the presence of more fatigue. Thus given that Test 7 has better performance than Test 6 despite being performed after Test 6 there is much stronger support for the argument that the hybrid control does indeed reduce the impact of FES-induced Fatigue. This is further backed up by the smaller final overall gain (k) for Test 6.

In general, the voltage threshold is expected to increase as more tests are conducted, however small reductions in fatigue can be observed during the tests in response to brief rests. Holding the reference arm at 0° for a while results in an increased response to the FES for the next movement with a larger response observed following longer rests (Fig. 5). However due to some minor mechanical issues which took time to repair (as described in Section “Results”), the rest time following Test 7 was longer than a few minutes thus allowing the arm more time to rest compared with the time between the other tests. Furthermore, the electrode position was not necessarily kept consistent due to the removal of electrodes and reapplication of electrode gel following Test 7. It is this increase in rest time that is the likely cause for the reduced voltage threshold seen for Test 8. Thus the values of the overall gain give a better comparison of the induced fatigue for each of the 6 min tests. It is also for these reasons that it is difficult to compare the results from Test 8 with the other FES tests although it is still useful to observe the parameter and error changes within Test 8. The RMSE values for each minute within all tests are shown in Fig. 9.

The first, solid blue bar for each test in Fig. 9, gives the RMSE for the overall test, while each of the following shaded bars gives the RMSE for each progressive minute, i.e. the first shaded bar gives the RMSE for the first minute, the second shaded bar gives the RMSE for the second minute and so on. For almost all tests the RMSE can be seen to improve from the first minute to the second although for most tests this is only by a small amount so could simply be attributed to differences in the reference movement or other small random variations. The two exceptions are the two motor only tests (Tests 9 and 10). The reasons for the results for Test 9 are discussed more thoroughly below. Test 10 does not perform assist-as-need so it is not expected that the performance would increase during the test and the change is only a few degrees so may be explained simply by differences in the reference movement or other small random variations. There is a larger decrease in the RMSE for some of the tests which start with larger estimates for the overall gain (Test 6 and 8) which is likely due to the adaptive nature of the control system, i.e. as the value for the overall gain becomes more accurate, the RMSE becomes smaller. However, the improvement for Test 7, while consistent over the first few minutes is not as significant despite also starting with an overall gain of 10, the same as Test 6 and 8. Overall it is expected that fatigue would cause an increase in RMSE over time in a FES system without adaptive parameters and assist-as-need. Indeed, during the last few minutes for the 6 min FES tests an increase in RMSE is observed which may be attributed to fatigue. Given that the increase and variability is larger for the FES only test (Test 6) than for the Hybrid test (Test 7) this further provides evidence for the hypothesis that hybrid exoskeletons can offer performance improvements over FES only systems with regards to precise control and fatigue reduction.

The Root Mean Square Error (RMSE) is the most commonly used measurement of performance for prosthesis and exoskeleton control systems [13] however given the early state of many of the upper-extremity hybrid exoskeletons described in previous works [6] very little statistical comparison can be made between the results from the hybrid exoskeleton described in this work and those described in [6]. Only one exoskeleton described in [6], The FES/Robot Hand, uses the RMSE as a measurement of performance [14, 15]. The tracking ability of the FES/Robot Hand was tested on 4 stroke subjects during 20 s long tests. The ability of the subject to track without the aid from the hybrid exoskeleton was compared to the tracking ability of the exoskeleton with different combinations of FES and motor support. The RMSE for the volitional movement was 10.9 degrees while the best exoskeleton performance (with a 50/50 balance of motor and FES support) was 4.9 degrees, and the RMSE for the FES only was about 8.5 degrees, resulting in an improvement in the RMSE of 6 degrees between no support and hybrid support, and an improvement of 3.6 degrees between FES only and the hybrid system.

It is important to note that the tracking tests for the FES/Robot hand were performed on the index finger which comparatively has a smaller range of motion as compared to the elbow joint so is it is difficult to directly compare to this work. Furthermore, the tests performed in this work involved a comparison between a healthy subject performing volitional movement and the same subject putting no effort in at all with FES and with the hybrid system, whereas the tests conducted on the FES/Robot Hand compared Stroke patients performing volitional movement with and without the help of the different exoskeleton systems [14, 15]. As this thesis tests the volitional movement of a healthy subject it is not expected that the exoskeleton would produce a reduction in error for this work. Furthermore, complete relaxation of a subject’s muscles is not always easy to achieve. In some cases a user may unintentionally fight or aid the FES. Thus, the focus of this work is to compare the performance of the FES on its own with that of the hybrid combination which, as given in Table 2, shows an improvement of RMSE of 13.2 degrees for a 6 min test (RMSE of 42.9 for the FES system compared with a RMSE of 29.7 for the hybrid system). While it is difficult to compare values directly both the results described in this thesis and the results described in [14, 15] demonstrate an improvement of the hybrid system over the use of FES on its own with regards to precision of movement.

One other exoskeleton described in [6], the Wearable Rehabilitation Robot [16], uses a similar type of performance measure. The Integral of the Square of the Error (ISE) is used to compare the performance of an exoskeleton with and without FES for movement of the shoulder and fingers. For these tests the power of the actuator was deliberately reduced below that which would normally be required. The performance of the system was found to be better when the FES was used in addition to the motor providing evidence that hybrid exoskeletons can reduce the power requirements of the actuator. This cannot be seen in the results presented in the current work as the power of the actuator was not limited in the same way.

A key novel contribution of the current work is to test whether the hybrid exoskeleton is able to reduce the level of FES-induced fatigue as this is something which has not been tested by the hybrid exoskeletons described in [6]. As has been described already this can be tested by comparing the variations in the final value of the overall gain (k). The greater reduction of the overall gain during the FES test compared to that of the later performed hybrid test indicates that the hybrid system is able to reduce the FES-induced fatigue.

Given that all of these tests were conducted on the same individual and same day it is expected that the final value for the overall gain would be of roughly similar magnitude, with some variation due to fatigue as described above. Thus it is promising to see that despite the large differences in the initial value of the overall gain, the final values are all a similar magnitude to one another, with one exception. The volitional test (Test 5) did not cause variations in the overall gain. This can be attributed to a lack of errors during the test which would normally cause the software to apply the FES. The value of the overall gain can only be updated if the FES has been applied a sufficient number of times. Given that Test 5 involved the subject moving both arm simultaneously there was very little error and thus very few reasons for the FES to be applied. This is not an issue from a control perspective as if the error were to increase then the FES would be applied and the overall gain would be calculated. Given that the user is very capable it is not a problem that the overall gain has yet to be calculated and from a patient monitoring perspective one can still observe that the FES input parameter values are small, desired assistance has decreased and remains sitting at 0% (Fig. 4), and yet error is also small. This strongly indicates a user which is capable of performing the movement completely on their own. The change in these values over time will also provide an indication regarding the user’s ability as the user becomes more fatigued and across several sessions. It is also promising to note that the exoskeleton does not appear to impede a user who is capable of fully performing the movement. Overall the assist-as-need with regards to the overall gain (k) performs well.

The assist-as-need of the motor is not quite as smooth as that of the overall gain which can be seen by comparing Test 9 and Test 10. It is expected that the desired assistance will fluctuate somewhat given that short rests can improve the effectiveness of the FES parameters, however the rate at which the desired assistance varies during these tests is faster and larger than is desirable. It’s possible that the rest angle of the right arm being greater than 0° contributes to this as well as the slow lowering speed of the motor. However, what is more likely is that the assumption made in Section “Desired Support”, with regards to Eq. 2, is a poor assumption. Equation 2 relies on the assumption that if a previous change in support results in a given change in error then applying that same change in support again would result in the same change in error. Based on the results, this is likely not the case. Generally it is not desirable to lower the arm too quickly though. Other improvements could be made by using the median angle error for a longer time period in addition to making changes to Eq. 2.

So far the hybrid exoskeleton described in this work has only been tested on one healthy subject. This is something which is a common issue with regards to current exoskeleton research in general. Furthermore, very few exoskeletons, and even less hybrid exoskeletons have been tested on stroke patients, let alone on large numbers of them. Cost is one of the main barriers to widespread testing of exoskeleton devices. The cost to construct the exoskeleton described in this work is very low (a few hundred NZD) which may help to lessen this barrier in future.

## Conclusion

Overall the hybrid control and assist-as-need control methods perform well in comparison with complete volitional movement and non-hybrid control. In particular the hybrid system shows an improved performance with regards to FES-induced fatigue compared with using FES only demonstrated by larger change in overall gain (k) and a larger average and RMSE error for the FES only control. As far as the authors are aware this is the first upper-extremity hybrid exoskeleton which uses model-based FES control to perform assist-as-need.

## Material and methods

The design of a voltage controlled functional electrical stimulator has been described in other works [17] and is the FES device used during the tests described in this work. It allows for control of a wide range of FES parameters. The electrodes used in this work are (50 mm × 50 mm) reusable e-textile electrodes, which have a similar performance and lower resistance than conventional hydrogel electrodes [18]. The exoskeleton in this work has been designed for the elbow joint.

For simplicity and portability the Rhino Motion Controls High Torque Servo Motor (RMCS-2251) has been selected as the actuator for this exoskeleton This motor is more than capable of providing all of the torque requirements for movement of the elbow joint [19]. A smaller and lighter motor could be used in place of this one in future. A portable and rechargeable Li-Po battery (Zippy, 4000 mAh, 11.1 V, Hardcase, 20C Series) was acquired for the supply for the system as a whole. It is combined with a 150 W adjustable boost circuit (purchased from prodctodc.com - Item ID #090438, set to 13.5 V for this system) and relay circuitry for added safety. The relay section of the circuit was constructed by one of the lab technicians. A 5 V regulator (L78s05cv) was used to step the 13.5 V down for the Arduino and motor. For testing described this work, a desktop DC power supply was used in place of a 3 V regulator for the input to the FES circuit as a slightly more consistent supply could be achieved. A 3 V regulator could be used instead for portability. Ethical approval for testing of this device was granted by the University of Canterbury Human Ethics Committee. Section “Exoskeleton Construction” and “Sensing” describe the construction of the exoskeleton and sensing system respectively.

### Exoskeleton construction

**Fig. 10:**
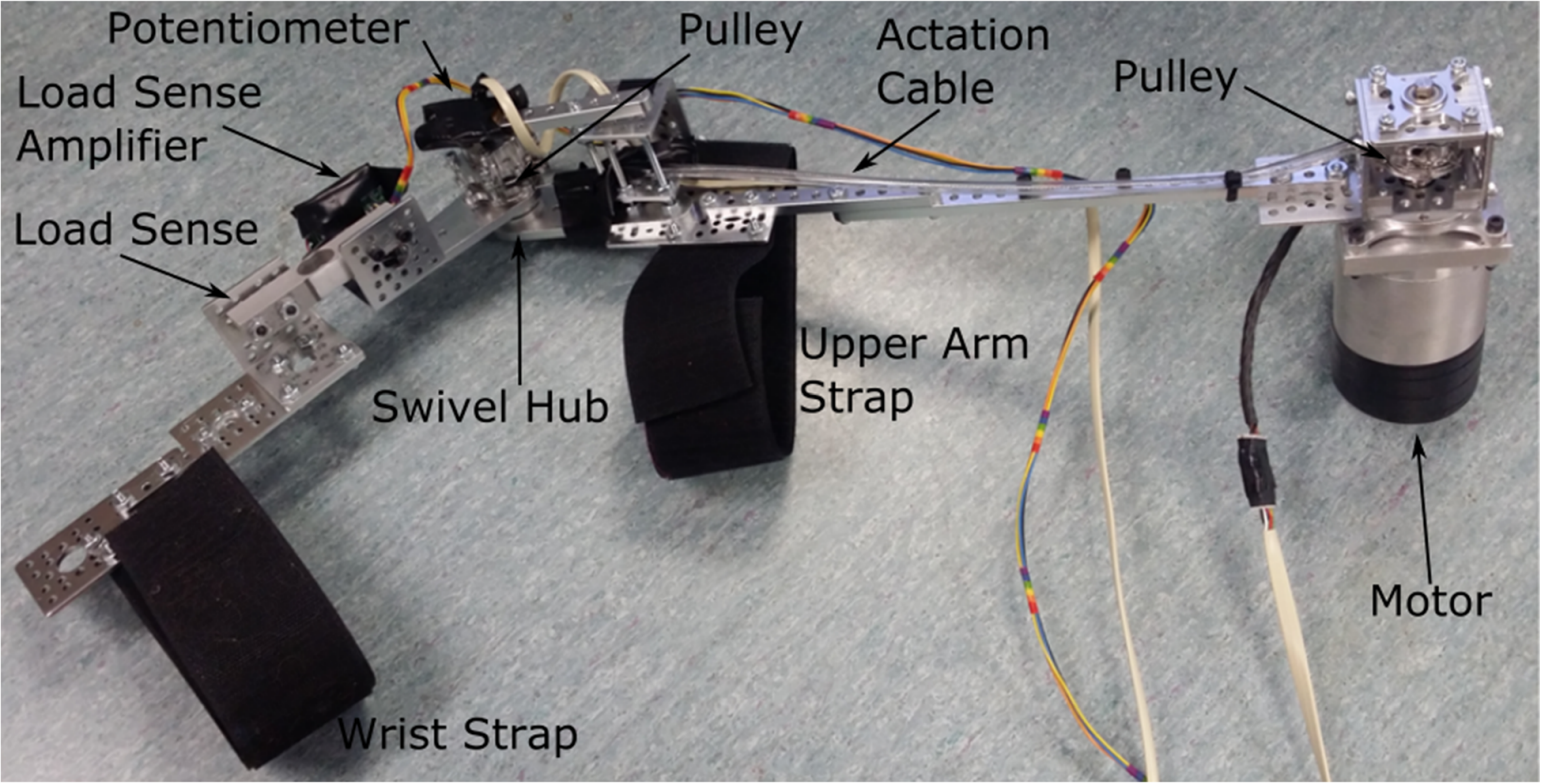
The Powered Exoskeleton (Right Arm)

**Fig. 11:**
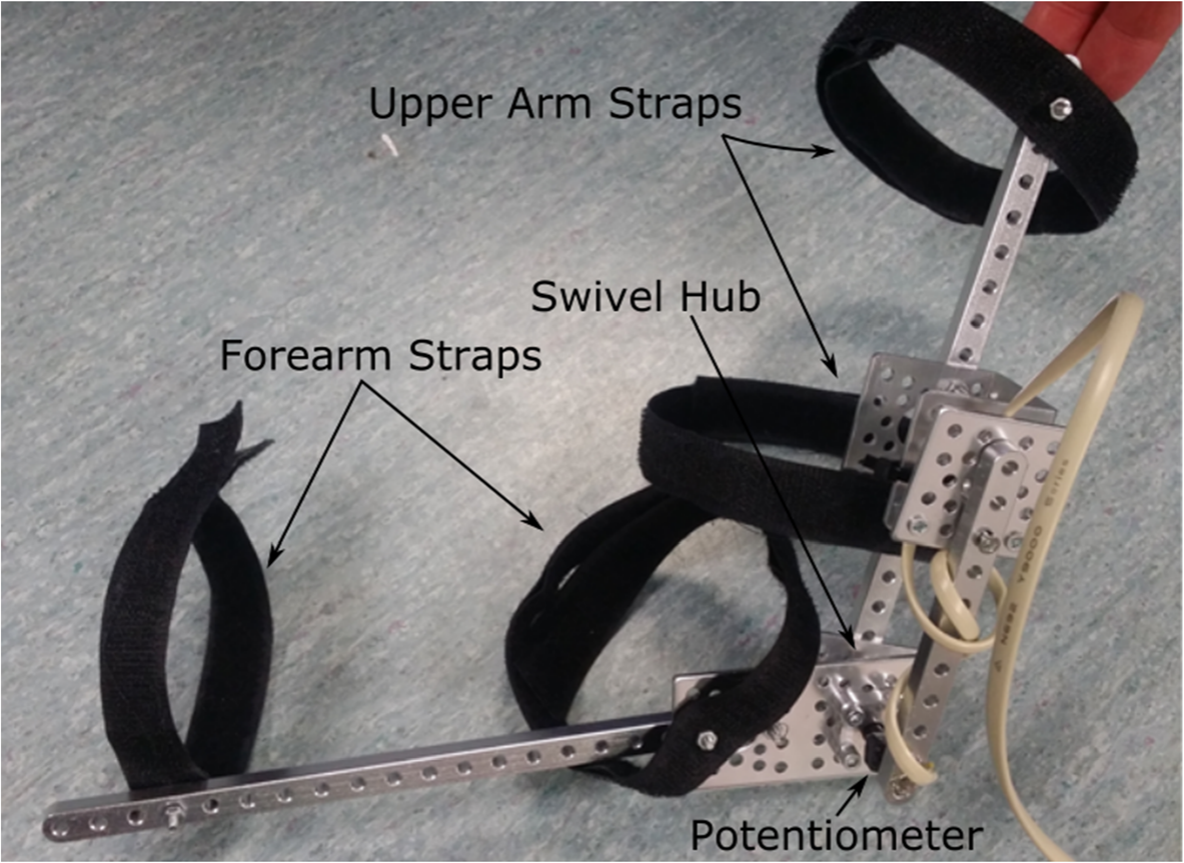
Unpowered Exoskeleton (Left Arm)

The powered exoskeleton was arbitrarily selected to be designed for the right-arm and a second non-powered smaller exoskeleton was designed for the left arm to be used as the control input. Construction of the both exoskeletons was based around Actobotics components sourced from Sparkfun [20]. The powered exoskeleton is shown in Fig. 10 and the unpowered exoskeleton is shown in Fig. 11.

A swivel hub allows free movement of the elbow joint for both exoskeletons. A pulley is affixed to one side of the swivel hub on the powered exoskeleton. A metal cable is wound around this pulley and runs up the inside of a protective plastic tube. At the other end of the plastic tube the cable is wound around a second pulley which is affixed to the shaft of the motor situated on the shoulder of the user.

**Fig. 12:**
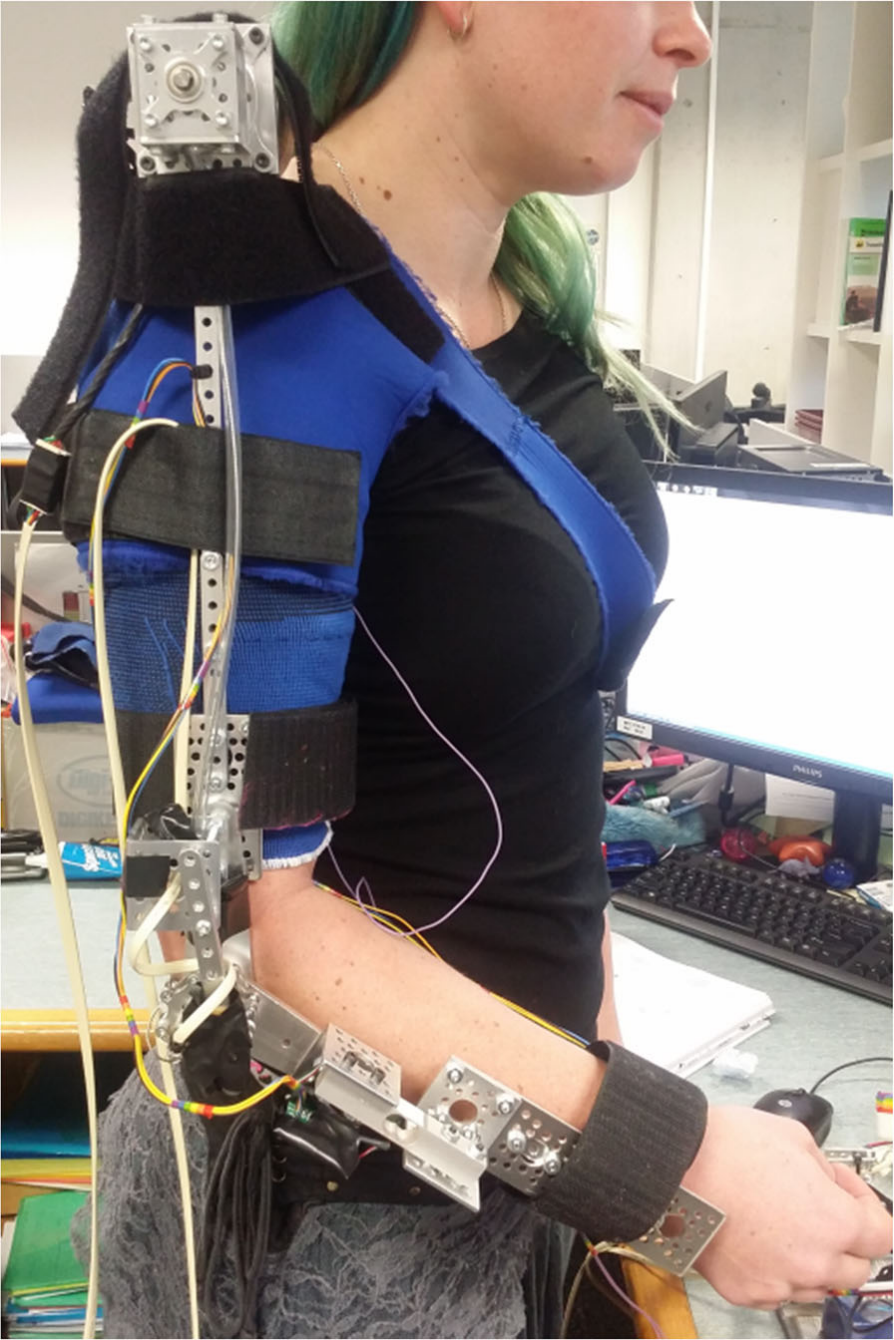
A User Wearing the Hybrid Exoskeleton

To attach the motor to the user a soft shoulder brace is worn. The exoskeleton is manually lined up with the user’s arm and the motor is placed gently on the shoulder. Velcro straps are used to hold the motor and exoskeleton in place. The FES sleeve is placed on the user’s arm and electrode gel (Spectra 360) is applied prior to attachment of the exoskeleton. Correct placement of the electrodes are also checked and any adjustments made prior to exoskeleton attachment. Figure 12 shows a user wearing the exoskeleton and FES sleeve.

The exoskeleton can be made shorter or longer for the shoulder to elbow section by unscrewing the upper metal rod and moving the screw up or down a hole. The entire attachment of the FES electrodes and exoskeleton to the user takes only 1–2 min and can be performed by the user themselves without the need for movement in the right arm. Despite the motor only weighing 180 g (and making up the bulk of the weight of the exoskeleton) the structure was still found to be heavy enough to cause mild discomfort during prolonged wearing (45 min) for a healthy subject. Future designs should consider methods to shift the weight of the motor to the centre of the back of the user and away from the shoulder.

### Sensing

**Fig. 13:**
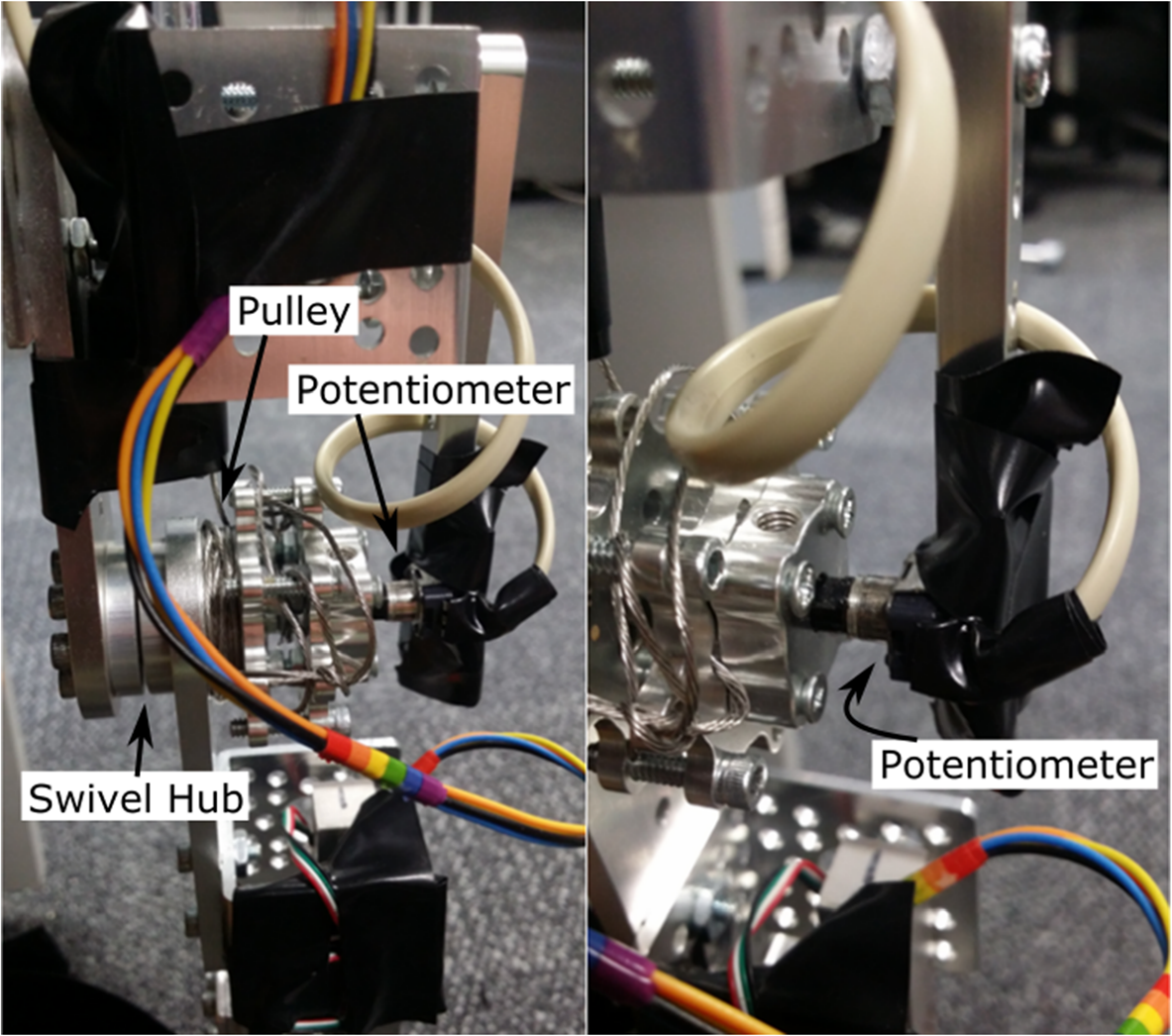
Elbow Joint with Potentiometer

To measure the angle of the arm, the shaft of a potentiometer was attached to the pulley at the elbow joint using a set screw hub (Actobotics) and the body of the potentiometer was soldered to a small Vero board and affixed to a rod. The rod is attached to the upper portion of the exoskeleton as shown in (Fig. 13). Thus by measuring the potentiometer voltage the angle can be calculated without regular recalibration. The same method is used to measure the angle of the unpowered left-arm exoskeleton.

**Fig. 14:**
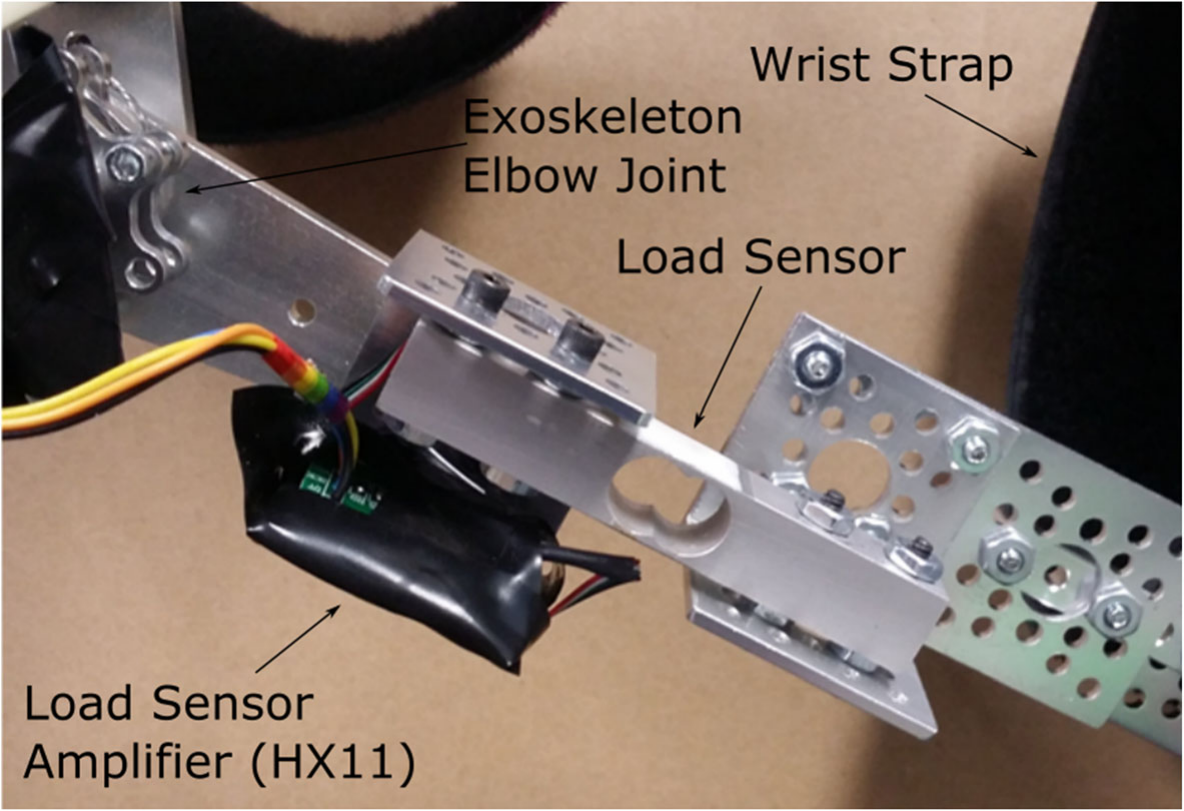
Connection of the Load Cell

**Fig. 15:**
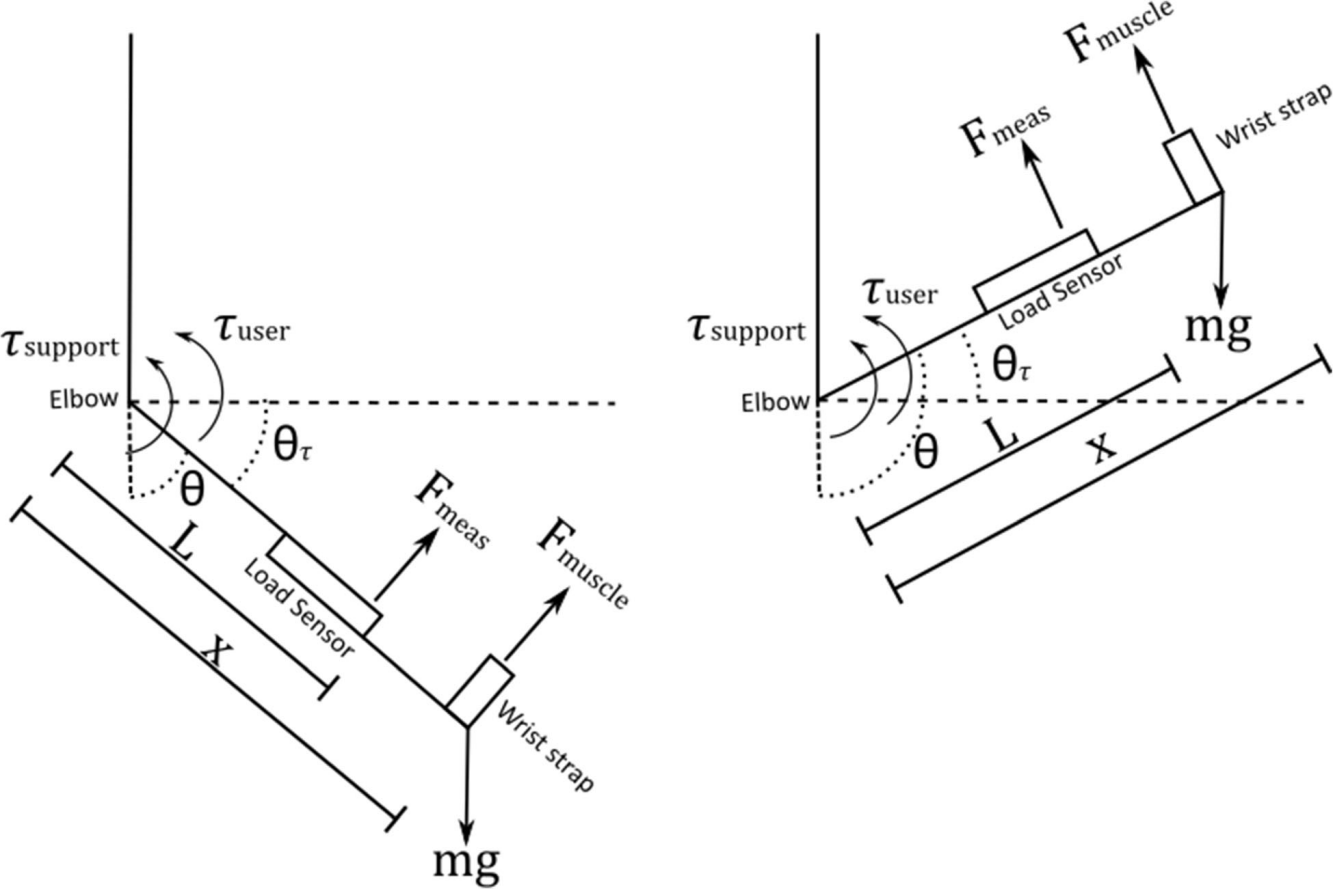
Free Body Diagram of the Exoskeleton Arm

To measure the force applied to and by the exoskeleton arm, a 10 kg straight bar load cell is used to connect the elbow section of the exoskeleton to the wrist section (Fig. 14). An HX711 load cell amplifier was used to interface between the load cell and the Arduino. The free body diagram of the exoskeleton is shown in Fig. 15.

The interaction forces between the user’s forearm and the exoskeleton all occur at the wrist strap point (x), which is located 0.2 m from the elbow pivot joint. The support forces from the motor are applied very close to the elbow pivot joint. The total torque applied at the exoskeleton elbow is the due to the torque produced from the interaction forces between the user and exoskeleton, and the support torque. The load sensor is located 0.13 m from the elbow pivot joint and measures the perpendicular force to the exoskeleton at this point. Thus the total torque at the exoskeleton elbow joint is the product of the force measured by the load sensor and the distance to the load sensor (0.13 m) as described by Eqs. 3 to 5. The force measured by the load sensor can be calculated from the load sensor reading using Eq. 6.

3 $$ {\tau}_{tot}={\tau}_{support}+{\tau}_{user} $$

4 $$ {\tau}_{user}=x\left({F}_{muscle}- mg\cos \left({\uptheta}_{\tau}\right)\right) $$

5 $$ {\tau}_{tot}=L{F}_{meas} $$

6 $$ {F}_{meas}= MR+c $$

Where:

*τ*_*tot*_ is the total torque around the exoskeleton elbow joint (anticlockwise).

*τ*_*support*_ is torque produced by the motor and cable system (anticlockwise).

*τ*_*user*_ is the torque produced by the user, includes volitional movement and effects from gravity (anticlockwise).

*x* is the distance from the elbow joint to the wrist strap (0.2 m).

*F*_*muscle*_ is the force produced by volitional movement from the user measured in Newtons (perpendicular to the arm and upwards).

*m* is the combined mass of the arm and exoskeleton in kilograms (at the wrist strap).

*g* is acceleration due to gravity (9.8 ms-^2^) (perpendicular to Earth and downwards)

θ is the elbow angle in degrees. This is the angle which the arm makes measured from 0° (when the arm hangs straight and perpendicular to Earth) in an anticlockwise direction.

θ_*τ*_ is (90 – θ) for arm angles below 90° and (θ – 90) for arm angles above 90°.

*L* is the distance from the elbow joint to the load sensor (0.13 m).

*F*_*meas*_ is the force measured by the load sensor in Newtons (perpendicular to the arm and upwards).

M is the gradient

c is the offset.

R is the output of the load sensor amplifier (volts).

Thus the measured total torque is given by Eq. 7.

7 $$ {\tau}_{tot}=L\left( MR+c\right) $$

The exoskeleton arm was rotated using the motor and cable system and measurements of the load sensor were taken at 14 different angles. This was repeated with three different weights attached to the end of the exoskeleton arm: 100 g, 200 g, and 500 g. Using the measurements and expected torque produced by each weight the gradient and offset in Eq. 6 were calculated. The mass of the empty exoskeleton arm was calculated to be 0.1026 kg.

Using these values, the expected torque produced by the mass of the exoskeleton can be calculated for a given angle using Eq. 4. This torque is defined as the set point for the given angle. If the torque calculated from the load sensor reading (Eq. 7) is greater (direction is anticlockwise and upwards) than the expected torque (Eq. 4) then the difference is due to the torque produced by the subject (either volitional or FES-induced) and furthermore the subject is supporting at least some of the weight of the exoskeleton arm in addition to their own. If the torque measured is equal to the set point then the subject is supporting their own arm weight but not the weight of the exoskeleton. This point is also called the 0% support point as well as the set point. If the torque measured is less than the set point then the exoskeleton is providing support for the subject.

In order to calculate the percent of support which the exoskeleton is providing, knowledge of the arm weight of the subject is required. During setup the system rotates the exoskeleton arm to 90° while the subject relaxes their arm. Several measurements are taken by the software and the results are averaged. From these measurements the arm mass of the subject can be calculated and the 100% support torque point is defined. Any torque measurements between this point and the set point mean that the exoskeleton is providing a certain percent support. For example half way between these two values would be 50% support.

## Data Availability

The datasets used and analysed during the current study are available from the corresponding author on reasonable request.

## Abbreviations

c: Offset of the load sense reading
f: Frequency in Hertz
FES: Functional Electrical Stimulation
ISE: Integral of the Square of the Error
k: Overall gain
M: Gradient of the load sense reading
PD: Proportional-Derivative
pw: Pulse-width in microseconds (full pulse length = positive + negative portions)
R: Output of the load sensor amplifier (volts)
RMSE: Root Mean Square Error
t: Current time
v: Voltage in volts
θ: Elbow angle in degrees
θτ: (90 – θ) for arm angles below 90˚ and (θ – 90) for arm angles above 90˚
*F*_*meas*_: Force measured by the load sensor in Newtons (perpendicular to the arm and upwards)
*F*_*muscle*_: Force produced by volitional movement from the user measured in Newtons (perpendicular to the arm and upwards)
*L*: Distance from the elbow joint to the load sensor (0.13 m)
$\overset{\sim }{e_{\theta }}$: Median angle error
$$ {e}_{\theta } $$: Angle error
f_*g*_: Frequency gain (equal to 0.22)
*g*: Acceleration due to gravity (9.8 ms‐²) (perpendicular to Earth and downwards)
*m*: Combined mass of the arm and exoskeleton in kilograms (at the wrist strap)
pw_*g*_: Pulse-width gain (equal to 0.15)
v_*g*_: Voltage gain (equal to 14)
x: Distance from the elbow joint to the wrist strap (0.2 m)
*τ*_*support*_: Torque produced by the motor and cable system (anticlockwise direction)
*τ*_*tot*_: Total torque around the exoskeleton elbow joint (anticlockwise direction)
*τ*_*user*_: Torque produced by the user, including volitional muscular movement and the effects from gravity (anticlockwise direction)
